# Application of the Scorpion Neurotoxin AaIT against Insect Pests

**DOI:** 10.3390/ijms20143467

**Published:** 2019-07-15

**Authors:** Sheng-Qun Deng, Jia-Ting Chen, Wen-Wen Li, Min Chen, Hong-Juan Peng

**Affiliations:** 1 Department of Pathogen Biology, Guangdong Provincial Key Laboratory of Tropical Disease Research, Southern Medical University, Guangzhou, Guangdong Province 510515, China; 2 Department of Anatomy, Histology and Embryology, Shanghai Medical School of Fudan University, Shanghai 200011, China

**Keywords:** AaIT, bioinsecticide, recombinant baculovirus, recombinant fungus, transgenic plants

## Abstract

*Androctonus australis* Hector insect toxin (AaIT), an insect-selective toxin, was identified in the venom of the scorpion *Androctonus australis*. The exclusive and specific target of the toxin is the voltage-gated sodium channels of the insect, resulting in fast excitatory paralysis and even death. Because of its strict toxic selectivity and high bioactivity, AaIT has been widely used in experiments exploring pest bio-control. Recombinant expression of AaIT in a baculovirus or a fungus can increase their virulence to insect pests and diseases vectors. Likewise, transgenic plants expressing AaIT have notable anti-insect activity. AaIT is an efficient toxin and has great potential to be used in the development of commercial insecticides.

## 1. Introduction

Insect pests cause serious damage in the agricultural field, which costs farmers billions of dollars annually [1]. In addition, vector-borne diseases, including malaria, result in more than one million human deaths every year [2]. Chemical insecticides are the most frequently and widely used in the control of agricultural pests and the vectors of human diseases [3,4]. However, continuous use of chemical insecticides creates a selective pressure that encourages the emergence of drug-resistant pests [5]. These excess chemical insecticide residues also contaminate the environment, including the soil and water, which is highly risky to humans and animals [6,7]. Biological pest-control offers a better alternative to chemical pest-control, because bioinsecticides, such as baculoviruses and entomopathogenic fungi, and have the advantages of targeting pests specifically, easy biodegradability, and being safe to both mammals and the environment [8,9,10,11]. In addition, breeding insect resistant cultivars such as *Bacillus thuringiensis* toxins recombinant cotton can partially resolve the pest problem in agriculture [12].

**Figure 1:**
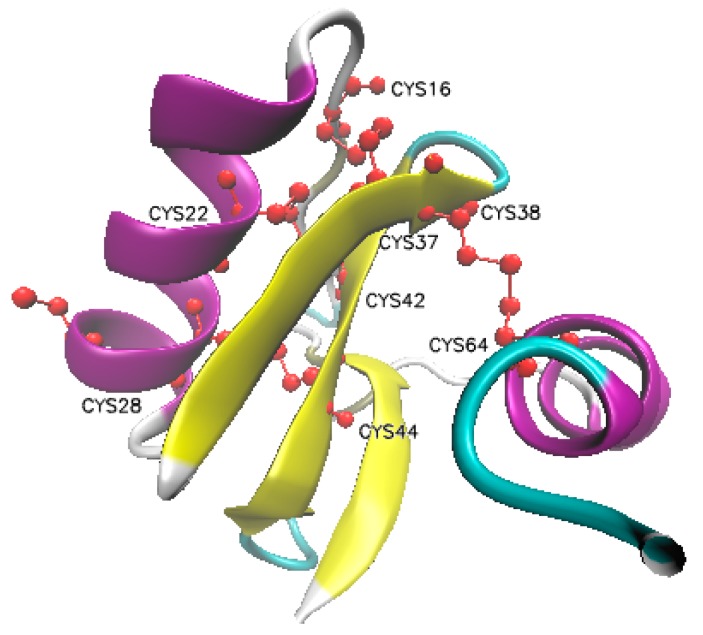
Structure of AaIT protein.

*Androctonus australis* Hector insect toxin (AaIT) is a single-chain neurotoxic polypeptide of 70 amino acids (Table 1) that was initially isolated from the venom of the buthid scorpion *Androctonus australis* Hector by Zlotkin et al. [13,14]. The amino acid backbone is cross-linked by four disulfide bonds, which are important for the structural stability and bioactivity of AaIT [15,16,17] (Figure 1). AaIT is specifically toxic to insects and has no effect on isopods and mammals, even at high doses [18,19]. This toxin exclusively targets insect voltage-gated sodium channels and modifies its gating mechanism [20,21]. AaIT binds to and excites the terminal of the insect’s motor nerve branches, stimulates the skeletal muscles massively and uncoordinately, causing fast excitatory paralysis and even the death of the insects [22,23]. Because of its strict selectivity and high bioactivity, AaIT has great potential to be used in the development of biological insecticides. Recombinant expression of AaIT in an insect-specific baculovirus or a fungus increases their toxicity to insects [24,25]. Moreover, the transgenic plants expressing AaIT have notable insect-resistant activity [23]. All of the information demonstrates that AaIT is a candidate neurotoxin to control insect pests if it can be efficiently delivered to the bodies of insect pests. In the current review, we summarize the development of the use of AaIT in insect pest control.

The ribbon diagram of the native structure of the AaIT toxin shows four disulfide bonds (marked in red) between residues of Cys16–Cys37, Cys22–Cys42, Cys28–Cys44, and Cys38–Cys64, respectively [21].

## 2. AaIT Recombinant Baculovirus Insecticides

Baculoviruses have been isolated from more than 400 insect species. Infection occurs following ingestion of virus-contaminated foliage by a susceptible host insect [26,27]. Depending on the virus–host combination and environmental conditions, certain kinds of baculoviruses, such as nuclear polyhedrosis viruses (NPVs), may take from five days to three weeks to kill the host insects [25]. Baculoviruses are insect-specific, environmentally safe, and pose no threat to human health, and show excellent compatibility with sustainable farming practices [28,29]. In addition, they are harmless to beneficial predators and parasitoids, as well as vertebrates and plants [30,31]. The commercial production of baculoviruses has been carried out in vivo, either by harvesting infected larvae from the field, or by infecting larvae reared on an artificial diet [25]. However, the relatively slow speed of killing the target pests is one of the major deterrents to their wide application as effective biocontrol agents [32]. 

To improve the insecticidal efficacy of baculovirus, *Bombyx mori* NPV was first genetically modified to express the AaIT toxin by Maeda et al. [19]. They found that when *Bombyx mori* larvae were infected with the recombinant virus, the AaIT toxin was secreted into the hemolymph and caused symptoms consistent with sodium channel blocking, which would reduce insect feeding damage and increase the rate of insect killing [19,33]. 

McCutchen et al. constructed a polyhedrin-positive *Autographa californica* NPV (AcNPV) expressing the AaIT protein [34]. They demonstrated that the 2nd instar larvae of *Heliothis virescens* that was infected by the recombinant baculovirus had a significantly shorter lethal time compared with that which was infected by the wild-type AcNPV [34]. Furthermore, it has been reported that a pyrethroid-resistant strain of *H*. *virescens* is more sensitive to this recombinant virus AcNPV-AaIT, compared to the wild-type AcNPV [35]. The first open-field release of a recombinant baculovirus carrying the AaIT gene occurred in the United States in 1995, consisting of an evaluation of AcNPV-AaIT for the control of *H. virescens* and *H. zea* on cotton [36]. The cotton trials showed that AcNPV-AaIT was significantly more effective in killing *H. virescens*, than *H. zea* [36,37]. In addition, Regev et al. found that the mortality of *Helicoverpa armigera* larvae was significantly increased by AcNPV-AaIT infection compared with the wild-type AcNPV infection [38]. Some field studies conducted on tobacco, lettuce, and cabbage, implied that foliar sprays of AcNPV-AaIT provided a higher efficacy in terms of the control of beet armyworm, *Spodoptera exigua* and cabbage looper, and *Trichoplusia ni* when compared with that of wild-type AcNPV [39]. In addition, when *B. thuringiensis* crystal protein gene (cry1–5) and the *AaIT* gene were introduced into the AcNPV genome, the infectivity and speed of action of this recombinant virus against the larvae of *Plutella xylostella* and *Spodoptera exigua* were dramatically improved compared with the wild-type virus [40,41]. Furthermore, it has been reported that some synergetic materials, such as fluorescent brightener 28 and Congo Red, promoted AcNPV-AaIT infection to *Spodoptera exigua* and *Pseudaletia separate* larvae [42,43,44]. 

In addition, the *AaIT* gene was also reported to be introduced into the genome of *H. zea* NPV to generate a recombinant virus (HzNPV-AaIT) [39]. Because of the host spectrum differences between the two viruses (HzNPV and AcNPV), HzNPV-AaIT killed larvae of both heliothine species (*H. zea* and *H. virescens*) at rates faster than AcNPV-AaIT did [39]. Furthermore, recombinant *Helicoverpa armigera* single-nucleocapsid NPV (HasNPV-AaIT) was more effective than the wild-type HasNPV in killing *Helicoverpa armigera* larvae [45,46,47,48,49,50]. Similarly, recombinant *Trichoplusia ni* NPV-AaIT could significantly inhibit the growth of *Trichoplusia ni* larvae and improve the killing efficacy toward the larvae compared with the wild-type virus [51].

To assess the effect of the recombinant virus on the non-target insects, two generalist predators, *Chysoperla carnea* Stephens and *Orius insidiosus* (Say) were fed on *H. virescens* larvae infected by AcNPV-AaIT, and the honey bee, *Apis mellifera* L., was injected with AcNPV-AaIT directly [52]. They found that the non-target insects were not adversely affected by the recombinant virus AcNPV-AaIT compared to the wild-type AcNPV [52]. Fuxa et al. found that when the 5th instar larvae of *Trichoplusia ni* were exposed to AcNPV-AaIT, only one of the 2484 second-generation insects was infected by this virus, which implied that vertical transmission would not happen to the non-target organisms contacting AcNPV-AaIT after field release [53]. Zhou et al. also revealed that HasNPV-AaIT exhibited significantly lower rates of the horizontal and vertical transmission than HasNPV in the field, which indicated that the recombinant virus will be transmitted at lower rates in *Helicoverpa armigera* populations than the wild-type virus [54]. Therefore, AaIT toxin expression in baculoviruses increases their virulence to target insects but has no adverse effects on non-target insects.

## 3. AaIT Recombinant Fungal Insecticides

Entomopathogenic fungi, such as *Beauveria bassiana* and *Metarhizium anisopliae*, have been used for the control of agricultural pests and the vectors of human diseases [55,56]. As environmentally friendly insecticides, entomopathogenic fungi are considered to be important alternatives or complements to chemical insecticides [4]. Wang and St Leger improved the pathogenicity of *M. anisopliae* by engineering it to express the neurotoxin AaIT [24]. They demonstrated that high-level expression of AaIT toxin in *M. anisopliae* resulted in a 22-fold increase in the fungal toxicity against larval *Manduca sexta* caterpillars and a 9-fold increase against adult *Aedes aegypti* [24]. Moreover, Pava-Ripoll et al. found that this recombinant fungus also possessed an increased virulence against coffee berry borer, *Hypothenemus hampei* larvae as compared with the wild-type strain [57]. Similarly, to improve the insecticidal efficacy of *B. bassiana*, the fungus was genetically modified to express the AaIT toxin (*Bb*-AaIT) [58,59]. Deng et al. indicated that *Bb*-AaIT had higher virulence than the wild-type strain in killing the larval and adult *Aedes albopictus* [58]. Likewise, Lu et al. suggested that *Bb*-AaIT had a faster action than wild-type fungus with regard to killing the larvae of the Masson pine caterpillar *Dendrolimus punctatus* and the wax moth, *Galleria mellonella* [59]. They also mentioned that when *B. bassiana* was genetically modified to express both the neurotoxin AaIT and insect cuticle-degrading protease PR1A (from *M. anisopliae*), AaIT could be degraded by the protease PR1A when they are expressed together [59]. However, recombinant *M. acridum* co-expressing Hybrid (the KCa/CaV blocker *ω*/*κ*-hexatoxin-Hv1a) and AaIT produced synergistic effect, requiring 45% fewer spores to kill half of the anopheline mosquitoes in five days than the single toxin strains [60]. Thus, a synergistic effect was evident in the fungus engineered with multiple toxin genes, while protein interactions need to be evaluated.

## 4. AaIT Transgenic Plants

The reduction of crops due to the damage by insect pests has caused huge losses to farmers [1]. Recently, the enormous potential of genome editing for pest control has been demonstrated in plants [12,61]. Breeding insect-resistant crop cultivars is the most environmentally benign and sustainable method for plants protection [62,63]. For example, crops expressing AaIT by genetic engineering have been developed and applied [23]. Yao et al. reported that the *AaIT* gene was introduced into the tobacco NC89 genome to produce the transgenic plant and most of the 2nd instar larvae of *H. armigera* died after feeding on the leaves of this recombinant tobacco, which implied that the transgenic tobacco had notable insect-resistant activity [64]. Moreover, Wu et al. demonstrated that transgenic hybrid poplars (*P. deltoides* × *P. simonii*) recombinated with the *AaIT* gene were significantly resistant to the 1st instar larvae of *Lymantria dispar*, compared with the wild-type plant [65]. It caused a decrease in leaf consumption by the larvae, a lower larval weight gain and higher larval mortality of *Lymantria dispar* [65]. In addition, Li et al. found that transgenic *Arabidopsis*, tobacco, and rice plants expressing the AaIT toxin are resistant to one chewing pest (*H. armigera*), two sucking pests (the whitefly and *Bemisia tabaci*), and the rice brown planthopper (*Nilaparvata lugens*), respectively [23]. They also constructed transgenic plants (*Arabidopsis*, tobacco, and rice plants) expressing both AaIT and snowdrop lectin (Galanthus nivalis agglutinin, (GNA)) [23]. These transgenic plants expressing both AaIT and GNA showed an increased resistance and toxicity to both chewing and sucking pests, when compared with the transgenic plants expressing either AaIT or GNA [23].

## 5. Other Application in Insecticide Development

Chilo iridescent virus (CIV), known as invertebrate iridescent virus 6, is the typical member of the genus *Iridovirus* within the family Iridoviridae [66]. CIV has a broad host spectrum, including numerous insect species of medical and agricultural importance [67]. Like baculovirus, CIV was recombinated with the AaIT gene in its genome to increase its virulence to insects [68]. It has been demonstrated that the CIV carrying the AaIT gene showed a strikingly enhanced ability in killing the 3^rd^ instar of *Galleria mellonella* larvae, compared with the wild-type CIV [68]. On the other hand, the lack of bioactivity of AaIT expressed in *Escherichia coli* due to the misfolded structure is also reported. Deng et al. stated that the suspension of the recombinant *Escherichia coli* expressing AaIT had little killing effect on *Aedes albopictus* and *Culex pipiens quinquefasciatus* larvae [17]. Similarly, Li et al. indicated that the *Pichia pastoris* recombinant with the AaIT gene was highly toxic to cockroach larvae, but the *E. coli* recombinant with AaIT was not toxic to cockroaches [69]. However, it has been reported that expression of a fusion protein of Cry1Ac and AaIT toxin in the acrystalliferous *B. thuringiensis* strain could improve its insecticidal activity against *Plutella xylostella* larvae [70].

## 6. Conclusions and Future Perspectives

Phenomenal advances in modern biology, in terms of tools and techniques, provide a good opportunity to develop genetic virulence and resistance against insect pests and vectors of human diseases. AaIT is toxic to many insect pests and disease vectors [22]. If AaIT can be efficiently delivered to the bodies of insect pests, it could enhance the virulence of both the recombinant viruses, and fungi and bacteria to insects, and increase the transgenic plants’ resistance against pests (Table 2). In addition, co-transformation of the *AaIT* gene and other toxin genes into one organism may further enhance its virulence, which may be a good strategy to increase the application of AaIT in insecticide development. In spite of these advantages, these recombinant organisms that stably express the exogenous toxins, might cause problems to the non-target organisms. Unfortunately, scientific and public aversion to field release of the genetically modified organisms has stymied the commercialization of these recombinant microorganisms and transgenic plants. Although the initial hypervirulent products should have features of biological containment, they should also be able to produce their second generation showing higher biosafety, and persisting longer in the environment, to provide sustainable cheap control for longer periods compared to the existing chemicals. For instance, a novel, highly virulent recombinant baculovirus was constructed to express AaIT and Cry1–5 under the control of an early promoter from *Cotesia plutellae* bracovirus [41]. This recombinant baculovirus expressing AaIT and Cry1–5 will revert to the wild-type genotype by serial passages in vitro, and therefore, it can be released into the environment with the guarantee of safety [41]. This is a new insight to improve the biosafety of the gene-modified insecticides. 

In summary, AaIT provides an effective tool for the design of new approaches to insect control. AaIT has great potential to be developed for and applied as a biological insecticide. However, before gene-modified microorganisms can be used in the field, more data on their effects on non-target organisms and the possibility of gene flow are required. Moreover, the precision and malleability of the molecular techniques also allow for the design of pathogens with different strategies for different ecosystems and avoiding resistance. If our understanding of the unique biology of genetically modified insecticidal microorganisms continues to grow and social acceptance is ensured through strict and transparent risk-benefit analysis, the field application of genetically modified insecticidal microorganisms will have a bright future.

## Figures and Tables

**Table 1:** Primary amino acid sequence for *Androctonus australis* Hector insect toxin (AaIT).

Protein	Amino Acids (70)
AaIT	KKNGYAVDSSGKAPECLLSNYCNNQCTCVHYADK GYCCLLSCYCFGLNDDKKVLEISDTRKSYCDTTIIN

**Table 2:** Application of AaIT against insect pests.

Category	New Strain Expressing AaIT	Test Insects	References
Recombinant baculovirus	*Bombyx mori *NPV-AaIT	*Bombyx mori* larvae	[19,33]
	*Autographa californica* NPV-AaIT	*Heliothis virescens*, *Heliothis zea, Spodoptera exigua*, *Trichoplusia ni*, and* Pseudaletia separate *larvae.	[34,35,36,37,38,39]
	*Heliothi zea *NPV-AaIT	*Heliothis virescens*, and *Heliothis zea *larvae	[39]
	*Helicoverpa armigera* single-nucleocapsid NPV-AaIT	*Helicoverpa armigera *larvae	[45,46,47,48,49,50]
	*Trichoplusia ni *NPV-AaIT	*Trichoplusia ni *larvae	[51]
	*Autographa californica* NPV-AaIT-Cry1–5	*Plutella xylostella *and* Spodoptera exigua *larvae	[40,41]
Recombinant fungus	*Metarhizium anisopliae*-AaIT	*Manduca sexta* caterpillars larvae, *Aedes aegypti *adults and* Hypothenemus hampei* larvae	[24,57]
	*Beauveria bassianae*-AaIT	*Dendrolimus punctatus *and* Galleria mellonella *larvae, *Aedes albopictus* larvae and adults	[58,59]
	*Beauveria bassianae*-AaIT-PR1A^#^	*Dendrolimus punctatus *and* Galleria mellonella *larvae	[59]
	*Metarhizium pingshaense*-AaIT	*Anopheles gambiae* larvae	[60]
	*Metarhizium pingshaense*-AaIT-Hybrid	*Anopheles gambiae* larvae	[60]
Transgenic plant	hybrid poplars (*P. deltoides* × *P. simonii*)-AaIT	*Lymantria dispar* larvae	[65]
	*Arabidopsis*-AaIT	*Helicoverpa armigera *larvae	[23]
	tobacco-AaIT	*Helicoverpa armigera* larvae*, Bemisia tabaci, *and* Nilaparvata lugens*	[23,64]
	rice-AaIT	*Bemisia tabaci, *and* Nilaparvata lugens*	[23]
	*Arabidopsis*-AaIT-GNA (Galanthus nivalis agglutinin)	*Helicoverpa armigera *larvae	[23]
	tobacco-AaIT-GNA	*Helicoverpa armigera* larvae*, Bemisia tabaci, *and* Nilaparvata lugens*	[23]
	rice-AaIT-GNA	*Bemisia tabaci, *and* Nilaparvata lugens*	[23]
Recombinat Chilo iridescent virus	Chilo iridescent virus-AaIT	*Galleria mellonella* larvae	[66]
Recombinant *Bacillus thuringiensis*	*Bacillus thuringiensis*-AaIT-Cry1Ac	*Plutella xylostella* larvae	[70]

All insect-specific viruses and fungi expressing *AaIT* exhibit higher virulence than their wild-type strains, and those microbes co-expressing *AaIT* with the other toxins are usually more toxic than the strains expressing *AaIT* alone, except that *Beauveria bassianae*-AaIT-PR1A has lower virulence than *Beauveria bassianae*-AaIT (labeled with #). The transgenic plants expressing *AaIT* are usually more resistant to insects than their wild-type controls, and the recombinant plants co-expressing *AaIT* with the other toxins are more resistant to insects than the strains expressing *AaIT* alone.

## Abbreviations

AaIT	Androctonus australis Hector insect toxin
GNA	Galanthus nivalis agglutinin
NPV	nuclear polyhedrosis virus (or nucleopolyhedrovirus)
AcNPV	Autographa californica (multiecupsid) nuclear polyhedrosis virus
HzNPV	Heliothis zea nuclear polyhedrosis virus
HasNPV	Heliothis armigera single-nucleocapsid nuclear polyhedrosis virus
CIV	Chilo iridescent virus
